# Laparoscopic appendectomy versus antibiotic treatment for acute appendicitis—a systematic review

**DOI:** 10.1007/s00384-021-03927-5

**Published:** 2021-04-14

**Authors:** Franziska Köhler, Anne Hendricks, Carolin Kastner, Sophie Müller, Kevin Boerner, Johanna C. Wagner, Johan F. Lock, Armin Wiegering

**Affiliations:** 1 Department of General, Visceral, Transplantation, Vascular and Pediatric Surgery, University Hospital, University of Wuerzburg, Oberduerrbacherstr. 6, 97080 Wuerzburg, Germany; 2 Department of Biochemistry and Molecular Biology, University of Wuerzburg, Wuerzburg, Germany; 3 Comprehensive Cancer Centre Mainfranken, University of Wuerzburg Medical Center, Josef-Schneiderstr. 2, 97080 Wuerzburg, Germany

**Keywords:** Acute appendicitis, Antibiotics, Laparoscopic appendectomy, Open appendectomy

## Abstract

**Background:**

Over the last years, laparoscopic appendectomy has progressively replaced open appendectomy and become the current gold standard treatment for suspected, uncomplicated appendicitis. At the same time, though, it is an ongoing discussion that antibiotic therapy can be an equivalent treatment for patients with uncomplicated appendicitis. The aim of this systematic review was to determine the safety and efficacy of antibiotic therapy and compare it to the laparoscopic appendectomy for acute, uncomplicated appendicitis.

**Methods:**

The PubMed database, Embase database, and Cochrane library were scanned for studies comparing laparoscopic appendectomy with antibiotic treatment. Two independent reviewers performed the study selection and data extraction. The primary endpoint was defined as successful treatment of appendicitis. Secondary endpoints were pain intensity, duration of hospitalization, absence from work, and incidence of complications.

**Results:**

No studies were found that exclusively compared laparoscopic appendectomy with antibiotic treatment for acute, uncomplicated appendicitis.

**Conclusions:**

To date, there are no studies comparing antibiotic treatment to laparoscopic appendectomy for patients with acute uncomplicated appendicitis, thus emphasizing the lack of evidence and need for further investigation.

## Introduction

Acute appendicitis (AA) is a common cause of acute abdominal pain worldwide, with an estimated lifetime risk of 7–8% [1, 2]. Typical symptoms are migration of initially periumbilical pain to the right lower quadrant of the abdomen or pain in the right iliac fossa, as well as rebound tenderness, fever, nausea, or vomiting. A shift in leucocyte count and a rise of C-reactive protein are often visible in laboratory tests. Transabdominal ultrasound, CT scan, or MRI of the abdomen are frequently used to verify the diagnosis [2].

Acute appendicitis is divided into different stages according to its severity, ranging from phlegmonous to perforated. Recent data suggest that appendicitis might not be a progressive disease but can be separated into “simple appendicitis” and “complex appendicitis.” Simple appendicitis is defined as suppurative or phlegmonous which rarely progresses into gangrenous or perforated appendicitis. In contrast, complex appendicitis is thought to progress quite rapidly to perforation or abscess [2].

Appendectomy is the standard treatment for AA and can be performed open or laparoscopically [3]. Studies have shown that laparoscopic appendectomy (LA) is superior to open appendectomy (OA) due to reduced pain intensity, wound infection rate, length of hospital stay, and enhanced quality of life following surgery [4].

Numerous trials and meta-analyses compared appendectomy to antibiotic treatment for AA. Overall, there was no significant difference in successful treatment between the two treatment options, except for a reduced absence from work favoring antibiotics. In the abovementioned studies, the majority of patients (80%) received open appendectomy. As LA has become the gold standard treatment for patients with suspected uncomplicated AA, we performed this systematic review to compare the outcome of antibiotic treatment and laparoscopic appendectomy.

## Methods

### Study selection and search strategy

We intended to include all types of studies with clinically and/or radiologically suspected AA that randomized patients over the age of 16 years to either laparoscopic appendectomy or antibiotic therapy. Studies investigating complicated appendicitis, immune-deficient patients, children, or pregnant women were excluded.

We searched PubMed database, Embase database, and Cochrane database on April 15, 2019, for studies meeting the abovementioned criteria. We included studies that were published between January 1, 1999, and April 15, 2019. Only studies with available full texts in German or English were considered. The MESH terms appendicitis, inflammation appendix, child, children, childhood, pediatric, and pediatrics were used in combination with AND, OR, and NOT. Outcomes of interest were defined as follows: length of hospital stay, duration of therapy, absence from work, overall mortality, overall morbidity, hospital readmission, treatment costs, and patient satisfaction. Outcomes regarding LA were defined as follows: perioperative blood loss, stump insufficiency, wound healing disorders, recurrent surgery, intraabdominal abscess.

Outcomes regarding antibiotic treatment were defined as follows: successful therapy without need of further intervention, necessity of appendectomy within 12 months after therapy, recurrent appendicitis within 12 months after therapy, side effects of antibiotics like diarrhea or anaphylaxis.

### Data collection

**Fig. 1:**
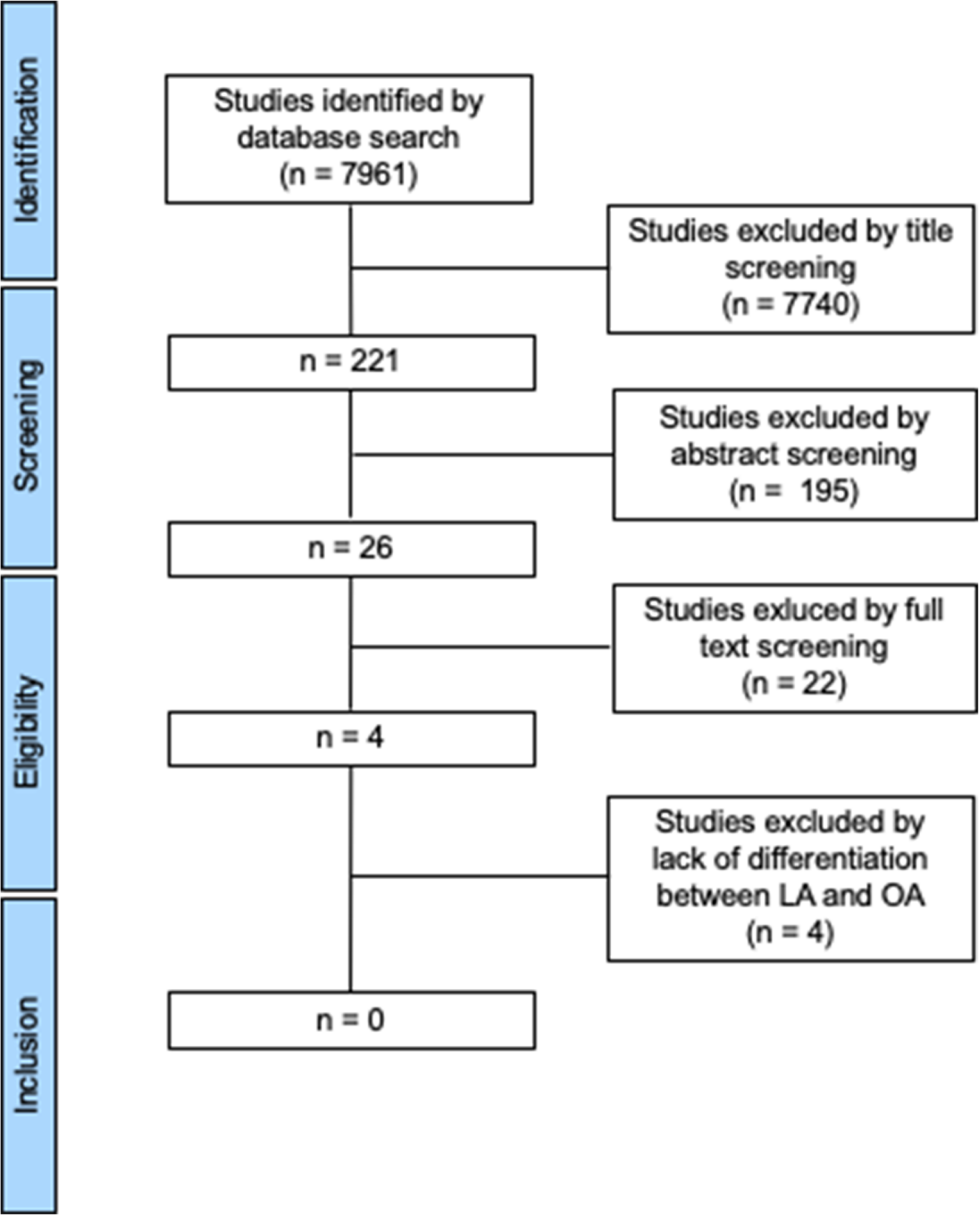
Selection of studies

We identified 7961 studies that were scanned for inclusion and exclusion criteria; the process is shown in Fig. 1. Very kindly some of the corresponding authors even provided their raw data to perform our review. Nevertheless, due to missing subgroup analysis for LA and OA considering our defined outcomes, we were not able to perform a meta-analysis.

## Results

By further investigation, no studies met our inclusion criteria. All considered studies did not differentiate outcomes between open and laparoscopic appendectomy. Thus, the intended meta-analysis comparing antibiotic therapy to laparoscopic appendectomy could not be performed.

## Discussion

Acute appendicitis is one of the most common causes of acute abdominal pain. Therefore, many studies have investigated its diagnostics and treatment.

Summarized in a 2018 published Cochrane review, Jaschinski et al. reported the advantages of LA over OA [4] and LA is currently the standard surgical procedure for the treatment of acute appendicitis [4–7]. Likewise, guidelines like the 2020 update of the WSES (World Society of Emergency Surgery) Jerusalem guidelines recommend LA for the treatment of acute appendicitis [8].

Another possible treatment for AA is the use of antibiotic agents to avoid surgical intervention. If conservative treatment is equal or superior to LA is still unanswered and without any clear recommendation. Therefore, the aim of this study was to further evaluate this question.

While performing our data search, we came across numerous studies comparing OA with antibiotic treatment. These studies reported inconclusive findings and superiority was proven for neither OA nor antibiotic treatment.

For left-sided diverticulitis, another inflammatory disease of the lower gastrointestinal tract, a paradigm shift took place over the last decade, showing that uncomplicated types can successfully be treated conservatively with antibiotics. Similar to acute appendicitis, antibiotic therapy is not standardized [9]. According to international as well as German guidelines, surgery is only needed in acute, complicated cases of diverticulitis, but can otherwise be performed after convalescence of the acute inflammation [10]. Considering this, conservative treatment could be reasonable in some stages of acute appendicitis.

Recently, “The CODA Collaborative” published their randomized trial comparing antibiotics with appendectomy including more than 1500 patients [11]. Primary outcome was defined as 30-day health status. The authors came to the conclusion that antibiotics are noninferior to appendectomy. Nevertheless, nearly 30% of patients treated with antibiotics needed appendectomy within the following 90 days. A total of 70% fewer surgeries due to acute appendicitis could relieve pressure on hospitals and conserve resources, which would be favorable in light of the COVID-19 pandemic. The majority of patients was able to be treated as outpatients and therefore sparing valuable hospital capacity. Even if 96% of appendectomies were performed laparoscopically in this study, there was no listing of particular complications regarding OA or LA. Furthermore, the study highlighted the presence or absence of appendicoliths but did not differentiate in complex or simple appendicitis. Overall, this well-researched study is another hint for the noninferiority of antibiotics in some cases of AA [11].

In summary, we were not able to find studies comparing laparoscopic appendectomy to antibiotic treatment for treatment of AA. Therefore, the question whether laparoscopic appendectomy is superior, comparable, or inferior to antibiotic treatment in patients with AA cannot be answered in this review. Considering the high incidence for acute appendicitis, this emphasizes the need for further broad studies to evaluate the primary question of superiority of either laparoscopic appendectomy or antibiotic treatment for acute appendicitis.

## Declarations

The funder had no role in study design, data collection and analysis, decision to publish, or preparation of the manuscript.
